# Apathetic Graves’ disease with severe hepatic and renal dysfunction induced by COVID-19 infection: Case report and literature review

**DOI:** 10.1097/MD.0000000000037456

**Published:** 2024-03-15

**Authors:** Liting Deng, Yingtong Zhang, Huilin Sun

**Affiliations:** The First Affiliated Hospital of Guangdong Pharmaceutical University, Guangzhou, Guangdong, People’s Republic of China.

**Keywords:** drug-induced liver injury, Graves’ disease, hyperthyroid-related liver injury, renal insufficiency

## Abstract

**Rationale:**

A rare and intractable case of apathetic Graves’ disease (GD) with severe liver and kidney damage induced by coronavirus disease 2019 (COVID-19) carries a certain risk of missing diagnosis and delayed treatment during the COVID-19 pandemic.

**Patient concern:**

A 60-year-old female patient developed anorexia, exhaustion, jaundice, nausea, and vomiting 10 days after COVID-19 infection. She was admitted to the Infectious Diseases Department because of recurring symptoms for more than a month.

**Diagnosis:**

Based on the patient’s epidemiological history, clinical symptoms, and prior history, she was preliminarily diagnosed with GD induced by COVID-19 with severe hyperthyroid-related liver injury and chronic kidney disease stage 4. Drug-induced and radiation-induced liver injuries occurred sequentially throughout the therapy.

**Intervention:**

Methimazole (MMI) (10 mg/d) was administered for 1 week, and the patient’s symptoms, thyroid function, and liver and kidney function improved. Nevertheless, the aforementioned symptoms and liver and kidney function deteriorated 20 days after increasing the MMI dose (20 mg/d). Therefore, in the presence of an artificial liver, hemodialysis, and other medical conditions, the treatment schedule was adjusted to individualized ^131^I anti-hyperthyroidism therapy.

**Outcome:**

After ^131^I treatment, the patient’s liver function returned to almost normal levels after a month, but worsened when the hepatoprotective drugs were stopped. Renal function did not deteriorate significantly and returned to baseline after 3 months. Thyroid function was restored to normal approximately 4 months later.

**Conclusion:**

COVID-19 may induce GD. Multidisciplinary collaboration can be initiated as early as possible. Individualized ^131^I therapy or long-term low-dose MMI (10 mg/d) can be considered to manage hyperthyroidism in GD patients with liver and kidney dysfunction and to prolong liver protection therapy appropriately.

## 1. Introduction

Graves’ disease (GD) is an autoimmune disease characterized by hyperthyroidism caused by the thyroid-stimulating hormone (TSH) receptor antibody (TRAb). It affects females more frequently and can be triggered by a high-iodine diet, infection, stress or pregnancy.^[1]^ Sympathetic nerve stimulation and hypermetabolism symptoms, primary clinical signs including palpitations, frequent bowel movements, weight loss, heat sensitivity etc.^[1]^ The prevalence in Chinese adults is 0.53%, and it decreases to 0.28% to 0.46% after the age of 60, which decreases with age.^[1,2]^ Apathetic hyperthyroidism is a unique and uncommon form of hyperthyroidism. It is a rare manifestation that primarily affects the elderly.^[2]^ It has an insidious onset, without sympathetic nervous excitation and hypermetabolism symptoms of typical hyperthyroidism, and shows nonspecific symptoms such as fatigue, poor appetite, vomiting, wasting, and lethargy. Additionally, patients are quite susceptible to arrhythmia, although their resting heart rate is <100 bpm. Physical signs of hyperthyroidism were either absent or atypical. It is easy to dismiss these symptoms as post-coronavirus disease 2019 (COVID-19) syndrome, which could postpone therapy. These clinical signs could be connected to the failure to promptly diagnose and treat hyperthyroidism.

Since the COVID-19 pandemic, studies have been conducted on some diseases and the aggravation of chronic diseases induced by COVID-19.^[3–5]^ Studies have also suggested that COVID-19 can cause immune-mediated inflammatory injury.^[3–5]^ In 2020, Bernard Equal conducted a large cohort study to follow-up on the changes in thyroid function data before and after COVID-19-infected patients. The majority of COVID-19 patients and survivors in the acute phase and follow-up showed no significant thyroid dysfunction, but there were a few reports of thyrotoxicosis and hypothyroidism in this study.^[6]^ The effect of the novel coronavirus on the thyroid gland remains clear.^[1,6]^ Researches on thyroiditis and GD after COVID-19 infection have also been reported subsequently.^[7,8]^ Herein, we present a case of GD with severe hepatic and renal dysfunction induced by COVID-19 infection. The patient has a complex personalized treatment process, and it is expected that this case can be used to explore alternative treatment options for GD patients with severe hepatic and renal dysfunctions. Written informed consent was obtained from the patient for publication of this case report and accompanying images.

## 2. Case presentation

A 60-year-old Chinese female was admitted to a reputable tertiary hospital in Guangzhou because of poor food intake, fatigue, nausea, and vomiting for more than 1 month. She had a 10-days history of COVID-19 infection before illness onset but no abnormal liver function history. At 53 days after onset, she experienced recurring symptoms, resulting in a weight loss of >5 kg. Upon physical examination, there was no evidence of GD orbitopathy and the thyroid gland was not enlarged. The lungs were clear to auscultate bilaterally; there was a normal sinus rhythm and no abdominal pain, and neurological examination indicated no tremors. Laboratory results of thyroid function were as follows: free triiodothyronine 13.60 pmol/L (reference range: 2.43–6.01), free thyroxine 50.50 pmol/L (reference range: 9.01–19.05), TSH <0.005 μIU/mL (reference range: 0.35–4.94), anti-thyroglobulin antibody 1047.00 IU/ml (upper limit of normal [ULN] 4.11), antithyroid peroxidase antibody 151.00 IU/ml (ULN 5.61), and TRAB 2.910 IU/L (ULN 1.75). The result of liver function and serum creatinine (SCR) were as follows: alanine aminotransferase (ALT) 203 U/L (ULN 40), aspartate aminotransferase (AST) 372 U/L (ULN 40), glutamyl transpeptidase 222 U/L (ULN 45), albumin 29.9 g/L (reference range: 35–50), total bilirubin (TBIL) 30.3 μmol/L (ULN 17.1), direct bilirubin (DBIL) 28.1 μmol/L (ULN 6.8), SCR 186.6 μmol/L (Reference range: 40–110) (Table 1). Color ultrasonography findings of the thyroid were indicative of alterations consistent with hyperthyroidism. Thyroid uptake and scanning revealed mild uniform enlargement of the gland, consistent with hyperthyroidism changes. Electrocardiography revealed paroxysmal atrial fibrillation.

**Table 1:** The result of liver function and SCR.

Date (d)	ALT (U/L)	AST (U/L)	TBIL (μmol/L)	DBIL (μmol/L)	GGT (U/L)	ALP (U/L)	SCR (μmol/L)
−75 (before COVID-19 infection)	18	18	2.8	1.1	44	159	193
53 (after illness onset)	203	372	30.3	28.1	222	197	186.6
80 (before inter-hospital transfer)	77	127	93.9	89.4	111	-	225
81 (MMI 10 mg/d started)	66	123	74.2	61.2	74	112	216
87 (MMI 20 mg/d started)	58	90	67.4	48.8	69	90	179
107 (MMI 20 mg/d)	72	464	89.7	59.2	352	204	197
110 (MMI 10 mg/d started)	82	465	143.7	101.1	205	155	-
114 (MMI 5 mg/d started)	67	243	154.5	107.9	138	143	-
118 (withdraw MMI)	50	160	143.9	102.4	144	160	237.0
120 (I^131^ treatment)	59	164	110.1	83.6	146	165	228.8
143	45	75	29.6	21.6	120	133	235.4
221	172	201	-	-	-	-	207.0

ALP = alkaline phosphatase, ALT = alanine aminotransferase, AST = aspartate aminotransferase, DBIL = direct bilirubin, GGT = glutamyl transpeptidase, MMI = methimazole, SCR = serum creatinine, TBIL = total bilirubin.

**Figure 1.**
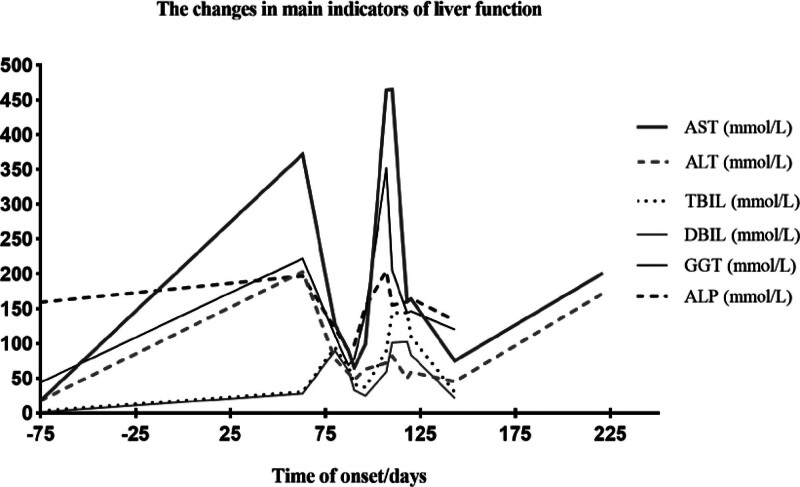
The changes of liver function of the patient. ALP = alkaline phosphatase, ALT = alanine aminotransferase, AST = aspartate aminotransferase, DBIL = direct bilirubin, GGT = glutamyl transpeptidase, TBIL = total bilirubin.

Once the patient was diagnosed with GD with hepatic and renal dysfunction, she received symptomatic medication to protect the kidneys and liver, as well as concurrent nutritional supplements and albumin, but without antithyroid drug (ATDs) therapy. More than 80 days after the onset of the disease, she complained of worsening nausea and vomiting, along with skin itching and increased sleep. Repeated laboratory tests showed persistently elevated TBIL and DBIL levels (Fig. 1). TBIL increased to 93.9 μmol/L, and that of DBIL increased to 89.4 μmol/L (Table 1). Simultaneously, blood ammonia increased to 101.3 μmol/L, and SCR increased to 225 μmol/L (Table 1). Consequently, she was transferred to the negative pressure intensive care unit of liver disease of another reputable hospital for further diagnosis and treatment 1 day later.

Before interhospital transfer, she had a history of hypertension and newly diagnosed type 2 diabetes mellitus but no history of viral hepatitis or allergy. More than 2 months prior to the COVID-19 infection, her SCR was 193 μmol/L, according to a search of her medical records. After interhospital transfer, a repeated physical examination revealed moderate skin and scleral jaundice and mild diffuse thyroid gland enlargement that was not visible but palpable, with no pain. The resting heart rate was 60 to 76 beats per minute, with sinus rhythm. Auxiliary examination after admission, 81 days after onset, showed worsening of liver function and SCR (Table 1). Repeated thyroid function revealed TT3 1.2 nmol/L, TT4 145.67 nmol/L, free triiodothyronine 4.79 pmol/L, free thyroxine 20.97 pmol/L, TSH < 0.0001 μIU/ml, TRAb 3.6 IU/ml, antithyroid peroxidase antibody 187.48 IU/ml. Furthermore, hepatitis virus serology was negative. Autoantibody profiles of autoimmune hepatitis (AIH): Antinuclear antibody, anti-liver cell membrane antibody, anti-smooth muscle antibody, anti-mitochondrial antibody, anti-mitochondrial antibody type M2, SP100 antibody, GP210 antibody, anti-hepatorenal microsomal antibody, anti-soluble liver antigen antibody, anti-hepatic cytoplasmic fluid 1 antibody, and anti-SS-B were all negative. Ceruloplasmin and IgG4 were negative. Indicators for tumors, including CEA and AFP, were within the normal ranges. Electrocardiography also indicated sinus rhythm. Two days later, the patient developed paroxysmal atrial fibrillation. Repeated thyroid ultrasound demonstrated a hypervascular gland consistent with GD but without apparent enlargement. Additionally, imaging revealed bilateral intralobular thyroid nodules, which were considered benign lesions. Abdominal ultrasound and CT tomography revealed thickening of the liver parenchymal echo, absence of the gallbladder following cholecystectomy, and mild-to-moderate ascites. No other abnormalities were observed. After excluding biliary obstruction and other common causes of liver injury, the patient was diagnosed with GD, hyperthyroid-related liver injury, and chronic kidney disease stage 4. The mean BWPS score was 30 during the course. ATD treatment was initiated at a low methimazole (MMI) dose of 10 mg/d. Subsequently, the patient’s symptoms improved, and liver and thyroid function improved (Fig. 1, Table 1). The MMI dosage was then increased to 20 mg/d after a week. Consistent symptomatic therapies, including liver protection, were administered during hospitalization.

The patient experienced recurrence of the aforementioned symptoms, which were more severe than their initial presentation, 20 days after the escalation of the MMI dosage. Reexamination showed that ALT did not significantly change during the future course of the disease, but AST and bilirubin did, with the highest AST, TBIL, and DBIL > 8ULN (Table 1). Since we believed that MMI was linked to drug-induced liver injury, we changed the dose to 5 mg/d. Subsequently, the reexamination of liver function showed elevated bile and reduced enzyme levels, which is characterized by “bile enzyme separation phenomenon,” and poor recovery after the withdrawal of MMI. Consequently, we believe that the present diagnosis of MMI-related drug-induced liver injury is clear. Two therapeutic schedules were selected after multidisciplinary discussion according to the relevant guidelines and consensus and the patient’s individual condition: A. If liver function continued to deteriorate, artificial liver therapy could be considered in the short term, and then return to low-dose MMI 10 mg/d to control hyperthyroidism for a long time. B. ^131^I treatment was selected for hyperthyroidism treatment. Short-term artificial liver auxiliary treatment may be administered to treat worsened liver injury after ^131^I treatment.

**Figure 2.**
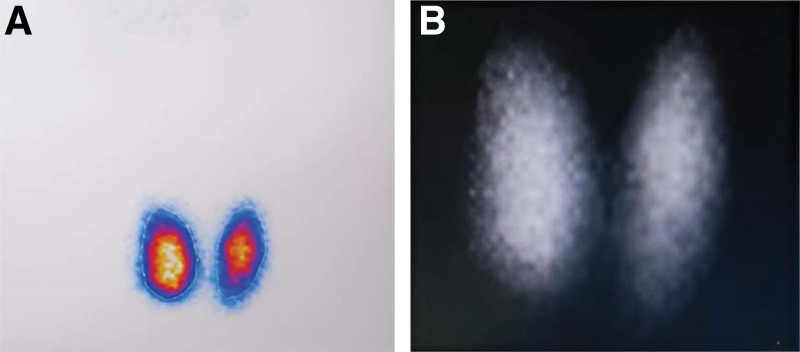
Thyroid technetium scanning of the patient.

Following a thorough discussion, multidisciplinary specialists provided comprehensive explanations to the patient and her family regarding the disease and the benefits and drawbacks associated with each therapeutic regimen, given the divergent approaches to the follow-up treatment. Treatment regimen B was ultimately chosen based on the patient’s wishes. Before ^131^I treatment, the thyroid technetium-99 m scan showed that the bilateral lobes of the thyroid were not enlarged, and the uptake was increased (Fig. 2). There was an increased thyroid iodine uptake rate (the 3 h was 17.6 % and the 24 h was 54.1%), which was still consistent with hyperthyroidism. The nuclear medicine department was asked to reduce the dose of ^131^I according to the individual condition of the patient. Liver, kidney, and thyroid functions were regularly monitored at outpatient endocrinology and liver disease clinics after ^131^I treatment. Following ^131^I treatment, there was no discernible decline in the liver or kidney function. Twenty-three days after ^131^I treatment, liver function showed mild liver injury, and with an SCR of 235.4 μmol/L, renal function did not substantially decline. A repeat electrocardiogram revealed sinus bradycardia. The patient stopped taking hepatoprotective drugs by herself approximately 30 days after ^131^I treatment. Together with subclinical hyperthyroidism, liver transaminase was raised by 4 ULN and SCR dropped to 207.0 μmol/L after 3 months. Thyroid function returned to normal levels approximately 4 months later.

## 3. Discussion

After a period of 10 days following the COVID-19 infection, the elderly woman developed apathetic signs associated with hyperthyroidism. A systematic review suggested that COVID-19 may induce autoimmune thyroid disease or exacerbate the underlying thyroid disease in remission.^[9]^ Evidence has shown that thyroid disease is strongly associated with COVID-19.^[7–10]^ GD development after COVID-19 infection is believed to take 6 to 8 weeks.^[10]^ However, in this case, it seemed that the patient’s GD developed in just 10 days. Transaminase and bilirubin levels were significantly elevated at the same time. Based on a previous study^[3]^: AIH should be considered in patients with liver damage after COVID-19 infection. When AIH and other liver diseases were excluded, the initial ATDs therapy was effective, confirming the diagnosis of hyperthyroid-related liver injury. However, owing to the elevation of the MMI dose, the liver function of the patient worsened. Despite the poor recovery following MMI dose decrease and cessation, liver function was better than it was previously, indicating MMI-related liver injury. After ^131^I treatment, liver function deteriorated after withdrawal of hepatoprotective drugs, indicating the possibility of radiation-induced liver injury. However, even though the cause of nephropathy is unknown, the diagnosis of chronic kidney disease stage 4 was considered based on SCR records.

Research findings indicate that 55% of individuals newly diagnosed with GD have abnormal liver function, of and the majority demonstrate mild to moderate liver injury.^[11]^ Currently, the cause of hyperthyroidism-induced liver injury is not completely clarified. According to a meta-analysis,^[12]^ the following are the primary potential mechanisms of hyperthyroidism-related liver injury: Long-term excessive transformation and metabolism of thyroid hormones increases the burden on the liver and leads to liver injury. In addition, thyroid hormones may have direct toxic effects on the liver. Hyperthyroidism can cause an increase in metabolic rate, resulting in a relatively hypoxic and malnourished liver following fatty degeneration, necrosis, liver fibrosis, and elevated ALT levels. Hyperthyroidism can lead to proliferation of Kupffer cells, an increase in AST secretion, and a decrease in reduced glutathione. Patients with GD may have elevated levels of TRAb and TSI, which could react with TSHR on the liver surface to trigger an immunological reaction that would damage the liver cell membrane structure. There is a positive correlation between the duration of hyperthyroidism and the occurrence and severity of hyperthyroidism-related liver injury.^[12]^ Thus, the goal of treating hyperthyroidism-related liver injury is to rapidly manage hyperthyroidism while also providing liver-protective therapy.^[2,11–14]^ In our case, ATD therapy was not initiated at the first hospital, which was thought to have been delayed.

Currently, only 3 treatments for GD are available. Given that each of the 3 approaches have its advantages and disadvantages, the clinical choice of treatment must be based on the patient’s desires and individual conditions. Apparently, surgery was inappropriate according to the patient’s condition. Some studies have compared liver function among different treatment schemes and found that the order of liver injury is MMI < propylthiouracil < radioactive iodine.^[12]^ Drug-induced liver injury is the most frequent adverse reaction associated with ATDs and often occurs within 3 months of treatment.^[2,13,15]^ propylthiouracil-related liver injury was not associated with the dose, which is mainly metabolized by the liver. Comparatively, MMI-related liver injury was positively correlated with medication dosage, primarily via the renal metabolism. Hepatocyte necrosis, intrahepatic cholestasis, and persistently aberrant liver function during liver metabolism are possible side effects of ATDs that can be alleviated by dosage reduction or withdrawal.^[2,13]^ Radioactive iodine mainly uses β-rays, which have a certain degree of ionization. After ^131^I treatment, the thyroid tissue is destroyed and a large amount of thyroid hormone is released for a short time, which has direct or indirect toxic effects on the liver, leading to or aggravating liver injury.^[12,16]^ Radioiodine is mainly excreted by the kidneys and renal insufficiency may cause unnecessary irradiation.

The patient was initially diagnosed with hyperthyroid-related liver injury complicated by paroxysmal atrial fibrillation. Research^[17]^ and guidelines^[2,13,16]^ suggested that ^131^I treatment can be given priority to GD with heart disease and hepatic insufficiency. Research^[11]^ and guidelines^[2]^ also recommend long-term drug therapy for patients with senile hyperthyroidism who are unwilling to receive ^131^I therapy. Moreover, it has been observed that 50% to 80% of those with abnormal liver function, up to fivefold above the ULN, can attain normal levels following the recovery of thyroid function by ATDs treatment.^[11]^ Before the ATD treatment, the patient refused ^131^I treatment; therefore, MMI therapy was initiated. Her medical condition improved during the course of the MMI therapy. However, an adverse event in the form of drug-induced liver injury manifested subsequent to the escalation of MMI dosage. In the case of drug-induced liver injury caused by ATDs, national and international guidelines and consensus suggest that it should be used with caution when transaminase elevation is 3ULN, and is contraindicated when transaminase elevation is >5ULN.^[2,13,14]^ In comparison with MMI-related liver injury, existing guidelines^[2,13,14]^ have no clear recommendations on the dose and adjustment time of MMI for hyperthyroidism-related liver injury. This lack of inclusion could be attributed to the infrequent incidence of severe liver injury due to hyperthyroidism. We had to titrate the dose from a small dose, even though the outcome did not meet our expectations. In our case, the hyperthyroid-related liver injury was effectively treated with a low dose of MMI (10 mg/d). We thought that when the patient’s liver function was significantly improved, routine GD treatment with high-dose ATDs could be started to control hyperthyroidism, and then the dose reduction process could be started. However, an increase in dose resulted in drug-induced liver damage. The timing and necessity of increasing the MMI dose after liver function improvement in this patient remain to be further explored. It remains unclear whether individualized ^131^I treatment after diagnosis can shorten the length of hospital stay. However, with a lower dose of ^131^I treatment, the patient’s hyperthyroidism did not transiently deteriorate, and her thyroid function returned to normal after more than 4 months. After the patient’s liver function returned to mild liver injury, the hepatoprotective drugs were discontinued. An increase in liver enzyme levels was detected in the outpatient clinic, which indicated radiation-induced liver injury. Research has recommended the use of hepatoprotective drugs for patients with elevated TBIL levels for 3 to 6 months after ^131^I treatment.^[18]^ Follow-up liver function was closely monitored if possible, reexamining weekly or at least monthly until liver function was normal.

In conclusion, when patients develop symptoms such as fatigue, loss of appetite, nausea, vomiting, and jaundice after COVID-19 infection, we should be aware of the possibility of apathetic hyperthyroidism combined with abnormal liver function. Multi-disciplinary cooperation should be initiated immediately. For patients with GD complicated with severe liver and kidney dysfunction, long-term low-dose MMI (10 mg/d) is recommended for anti-hyperthyroidism treatment, supplemented by liver-and kidney-protective drugs. The use of ^131^I therapy should consider the possibility of aggravating the liver dysfunction. With multidisciplinary cooperation, the ^131^I treatment dose can be calculated individually and the duration of liver protection treatment can be extended. In cases where liver and kidney function deteriorate to the point of failure or a thyroid storm develops, it is imperative to promptly administer artificial liver support and hemodialysis. In addition, close communication with patients should be conducted to ensure the effective implementation of treatment.

## Author contributions

**Conceptualization:** Liting Deng.

**Data curation:** Yingtong Zhang.

**Formal analysis:** Yingtong Zhang.

**Supervision:** Huilin Sun.

**Validation:** Huilin Sun.

**Writing – original draft:** Liting Deng.

**Writing – review & editing:** Liting Deng, Huilin Sun.
